# Supplementary material for: Hepatic SerpinA1 improves energy and glucose metabolism through regulation of preadipocyte proliferation and UCP1 expression
Source: Nat Commun. 2024 Nov 12;15:9585. doi: 10.1038/s41467-024-53835-9 (PMC11557585; doi:10.1038/s41467-024-53835-9)
Supplement: Supplementary file 1 — Supplementary Information [file 41467_2024_53835_MOESM1_ESM.pdf]

Supplementary Information

Hepatic SerpinA1 Improves Energy and Glucose Metabolism through Regulation of Preadipocyte Proliferation and UCP1 Expression.

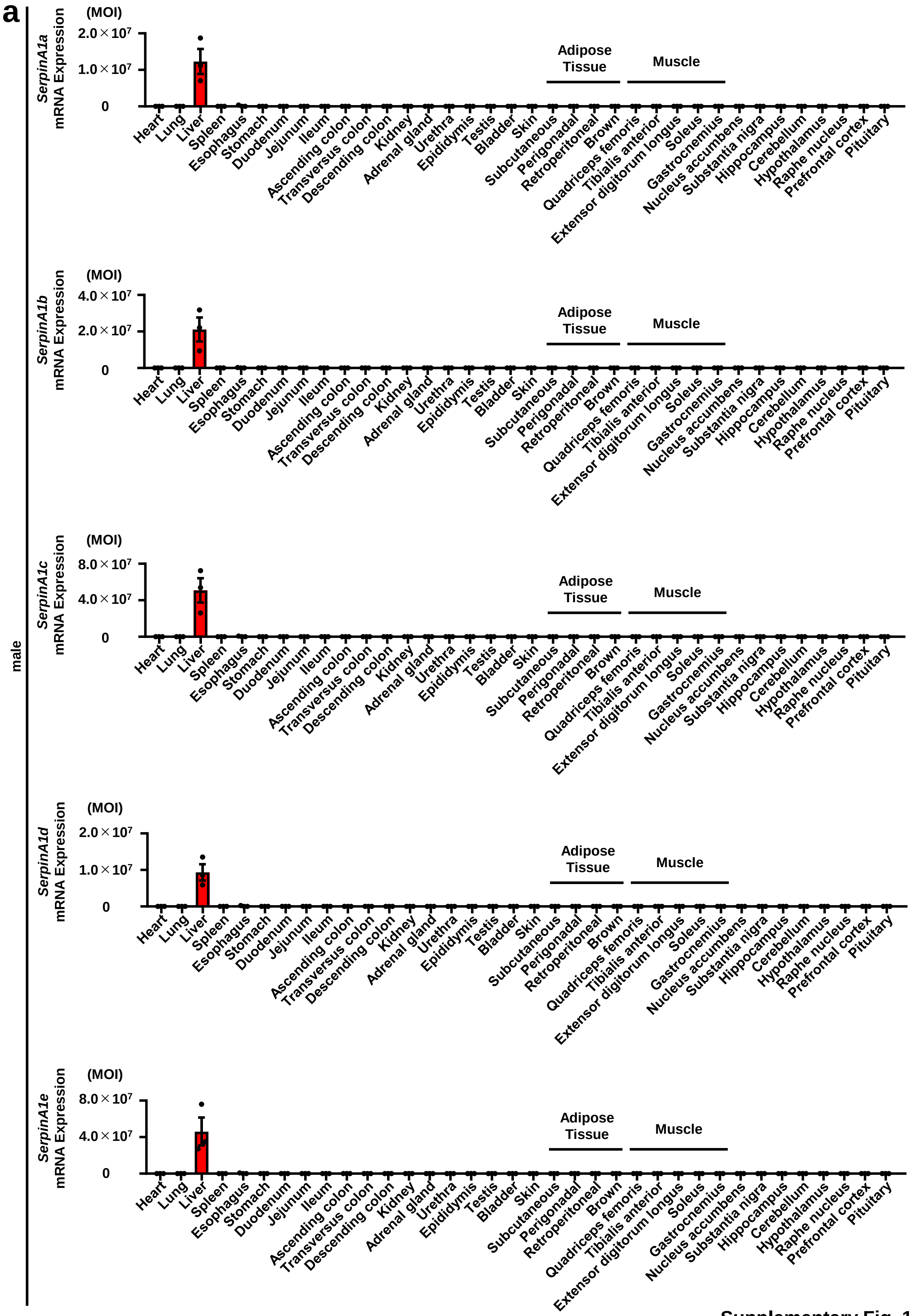

Supplementary Fig. 1

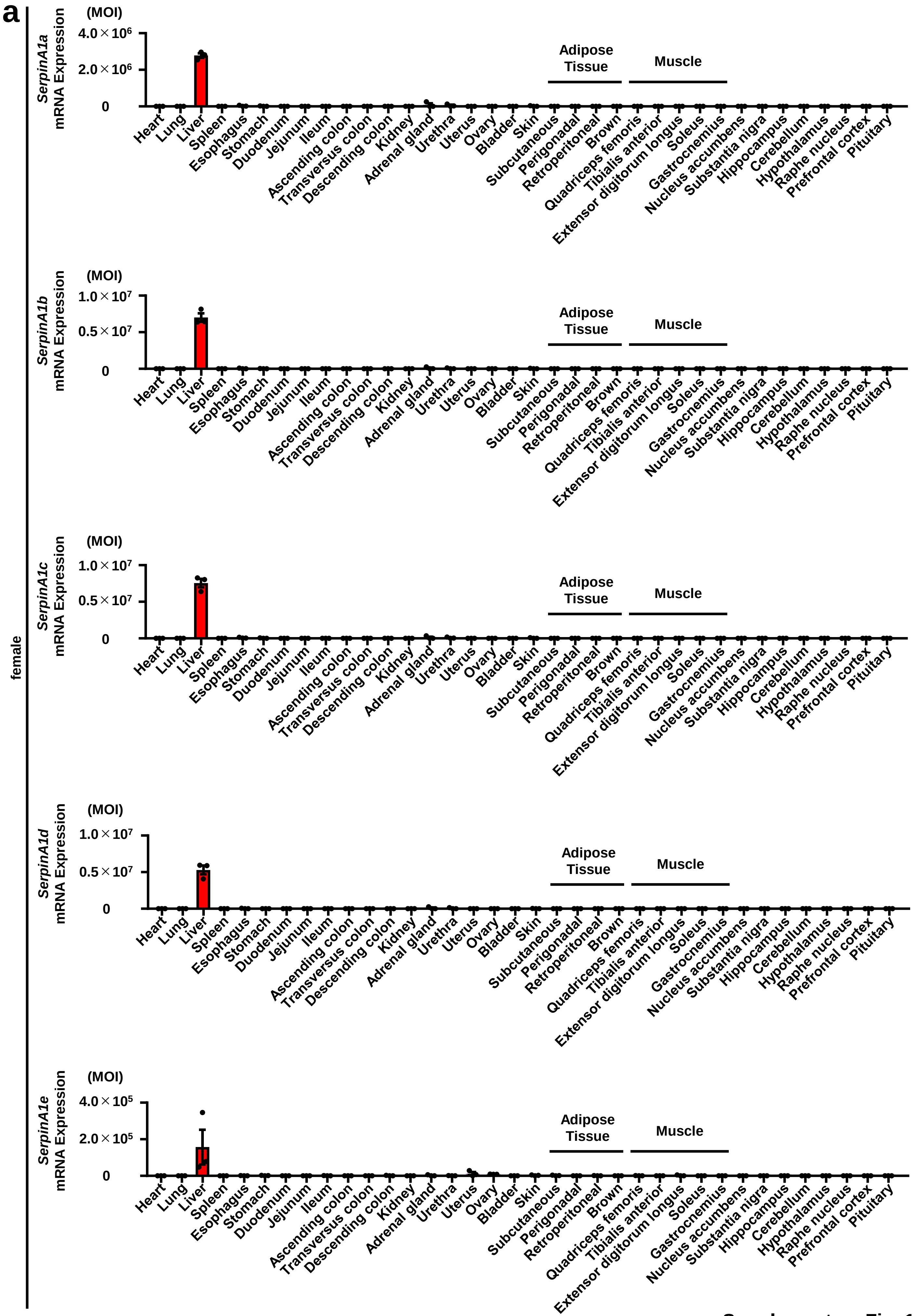

Supplementary Fig. 1

Supplementary Fig. 1 SerpinA1 induces preadipocyte proliferation

(a): *SerpinA1* mRNA expression in various organs and tissues of 4-month-old male and female C57BL/6 mice with primers that recognize individual SerpinA1 paralogs. The data are presented as the mean ± SEM (n = 3).
Source data are provided as a Source Data file.

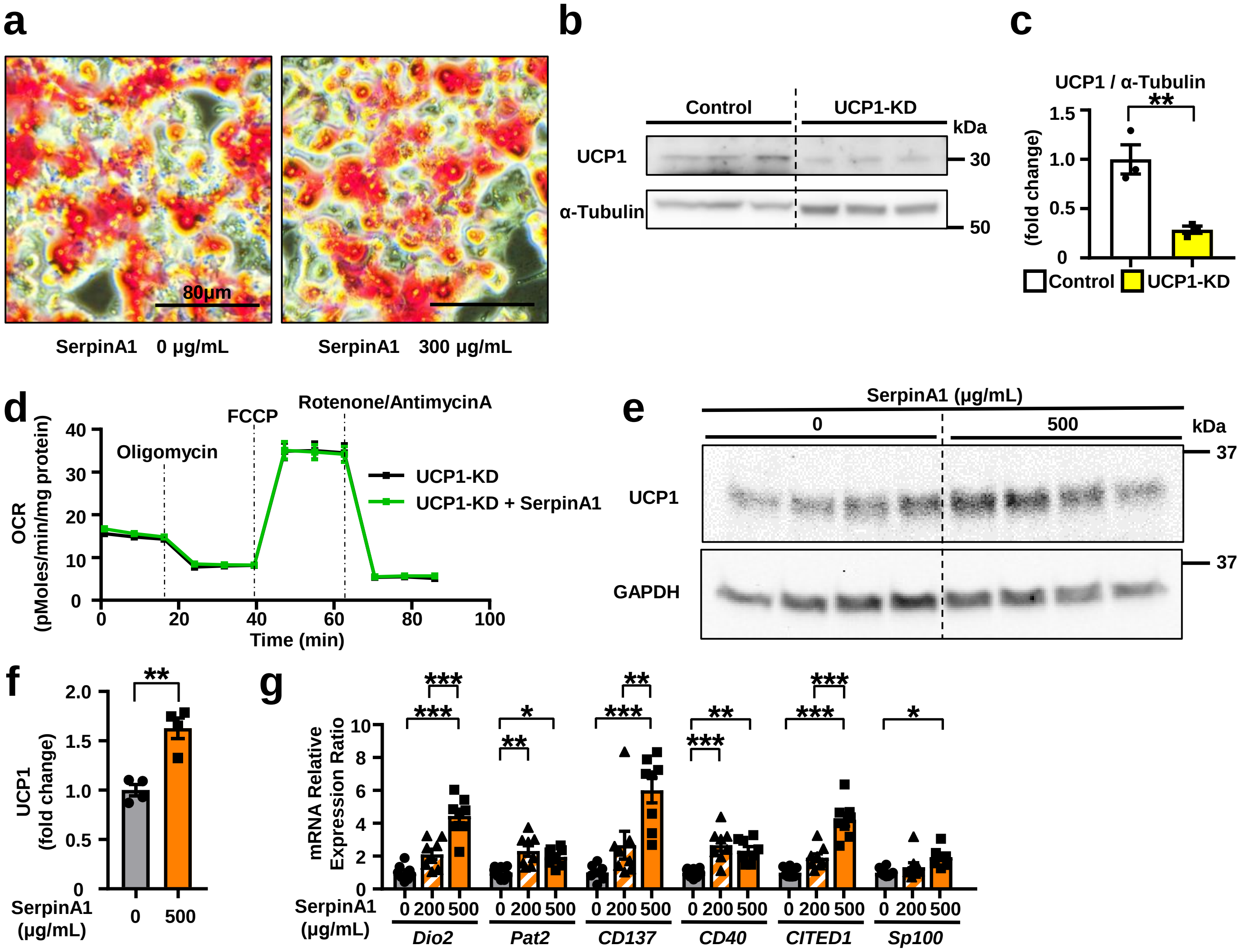

Supplementary Fig. 2 SerpinA1 induces mitochondrial activity in both brown and white adipocytes

(a): Oil Red O staining of mature brown adipocytes treated with different concentrations of SerpinA1 (0 or 300 μg/mL) for 24 h. The experiments were repeated at least three times independently. Scale bar = 80 μm.
(b): Immunoblotting of UCP1 in lysates from control and UCP1-KD mature brown adipocytes (n = 3).
(c): Quantification of UCP1 protein levels in (b), expressed relative to the level of the protein standard. The data are presented as the mean ± SEM (n = 3 biologically independent cell clones/group, two-tailed Student's t test, ***p* = 0.0095).
(d): Representative traces of the oxygen consumption rate (OCR) in UCP1-KD mature brown adipocytes treated with 0 or 300 μg/mL SerpinA1 for 16 h. The data are presented as the mean ± SEM (n = 10 biologically independent cell clones/group).
(e): UCP1 protein expression in mature white adipocytes treated with 0 or 500 μg/mL SerpinA1 for 36 h (n = 4).
(f): Quantification of UCP1 protein levels in (e), expressed relative to the level of the protein standard. The data are presented as the mean ± SEM (n = 4 technical replicates/group, two-tailed Student's t test, ***p* = 0.0019).
(g): Relative mRNA expression of genes in mature white adipocytes treated with different concentrations of SerpinA1 (0, 200, or 500 μg/mL) for 16 h. The data are presented as the mean ± SEM (n = 8 technical replicates/group, one-way ANOVA post hoc Bonferroni test, **p* < 0.05, ***p* < 0.01 and ****p* < 0.001).
Source data are provided as a Source Data file.

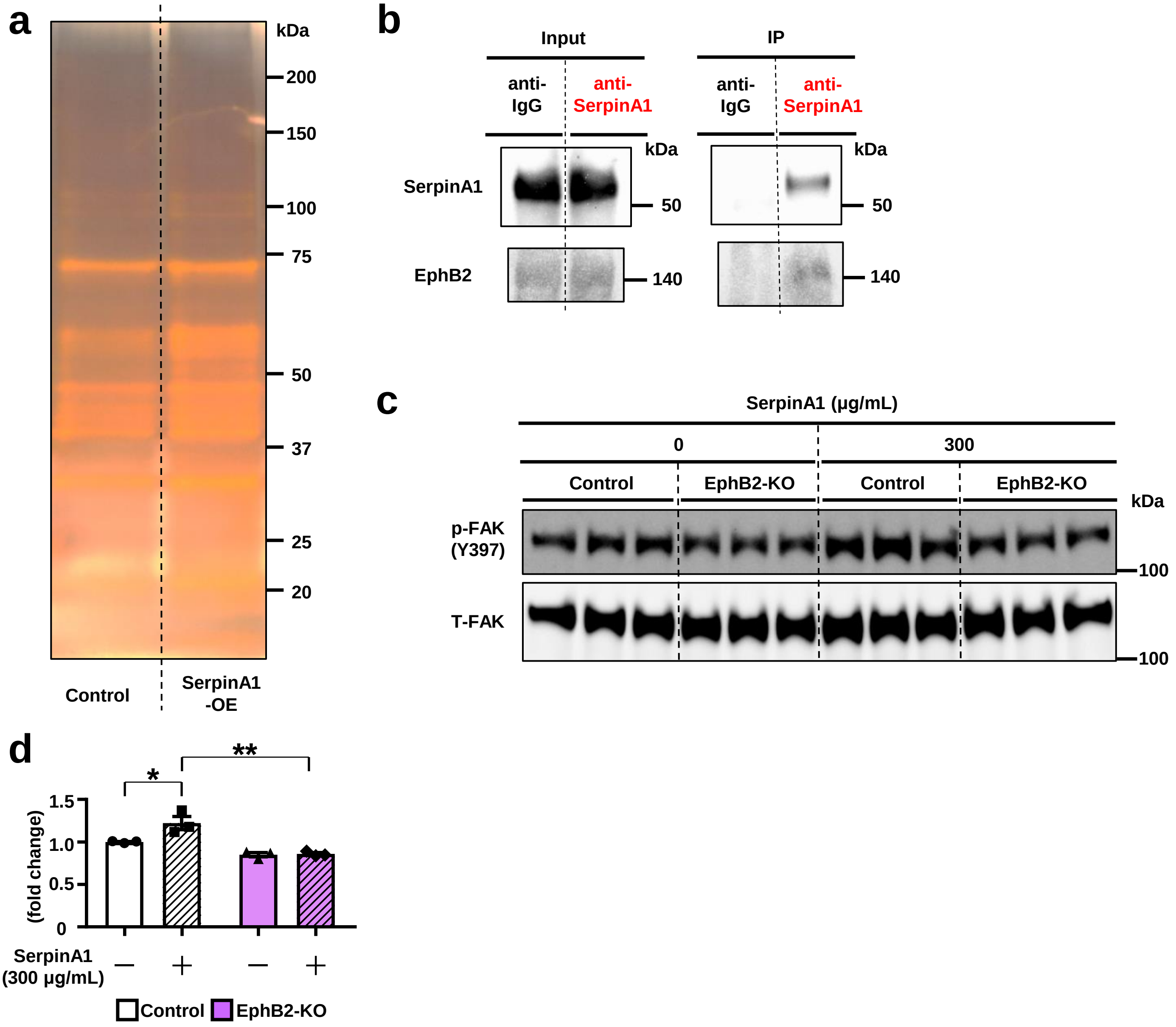

Supplementary Fig. 3 SerpinA1 forms a complex with EphB2 to promote preadipocyte proliferation

(a): Silver staining of post-IP samples (Control and SerpinA1-OE) separated by SDS–PAGE, for searching the SerpinA1 complex. The post-IP samples were prepared brown preadipocytes from the control group and the adenovirus-mediated 3×Flag-tagged SerpinA1-OE group.
(b): Immunoblotting of SerpinA1 and EphB2 in lysates before and after immunoprecipitation of mice liver and BAT composite samples with anti-SerpinA1 antibody, using anti-IgG antibody as control. (n = 1).
(c): Immunoblotting of p-FAK in lysates from control and EphB2-KO brown preadipocytes treated with different concentrations of SerpinA1 (0 or 300 μg/mL, 24 h) (n = 3).
(d): Quantification of p-FAK protein levels in (c), expressed relative to the T-FAK protein level. The data are presented as the mean ± SEM (n = 3 biologically independent cell clones/group, one-way ANOVA post hoc Bonferroni test, *$p < 0.05$ and **$p < 0.01$).
Source data are provided as a Source Data file.

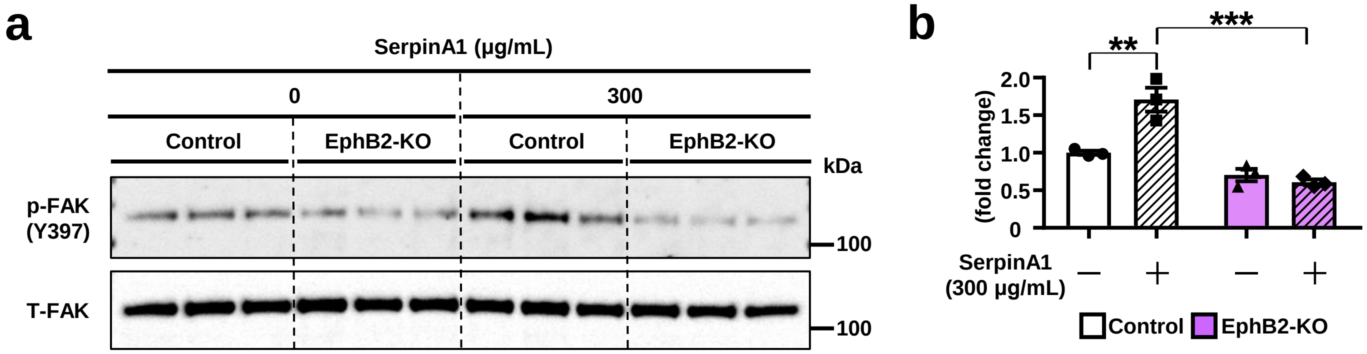

Supplementary Fig. 4 SerpinA1 induces adipocyte browning through interaction with EphB2

(a): Immunoblotting of p-FAK in lysates from control and EphB2-KO mature brown adipocytes treated with different concentrations of SerpinA1 (0 or 300 μg/mL, 24 h) (n = 3). (b): Quantification of p-FAK protein levels in (a), expressed relative to the T-FAK protein level. The data are presented as the mean ± SEM (n = 3 biologically independent cell clones/group, one-way ANOVA post hoc Bonferroni test, $^{**}p < 0.01$ and $^{***}p < 0.001$). Source data are provided as a Source Data file.

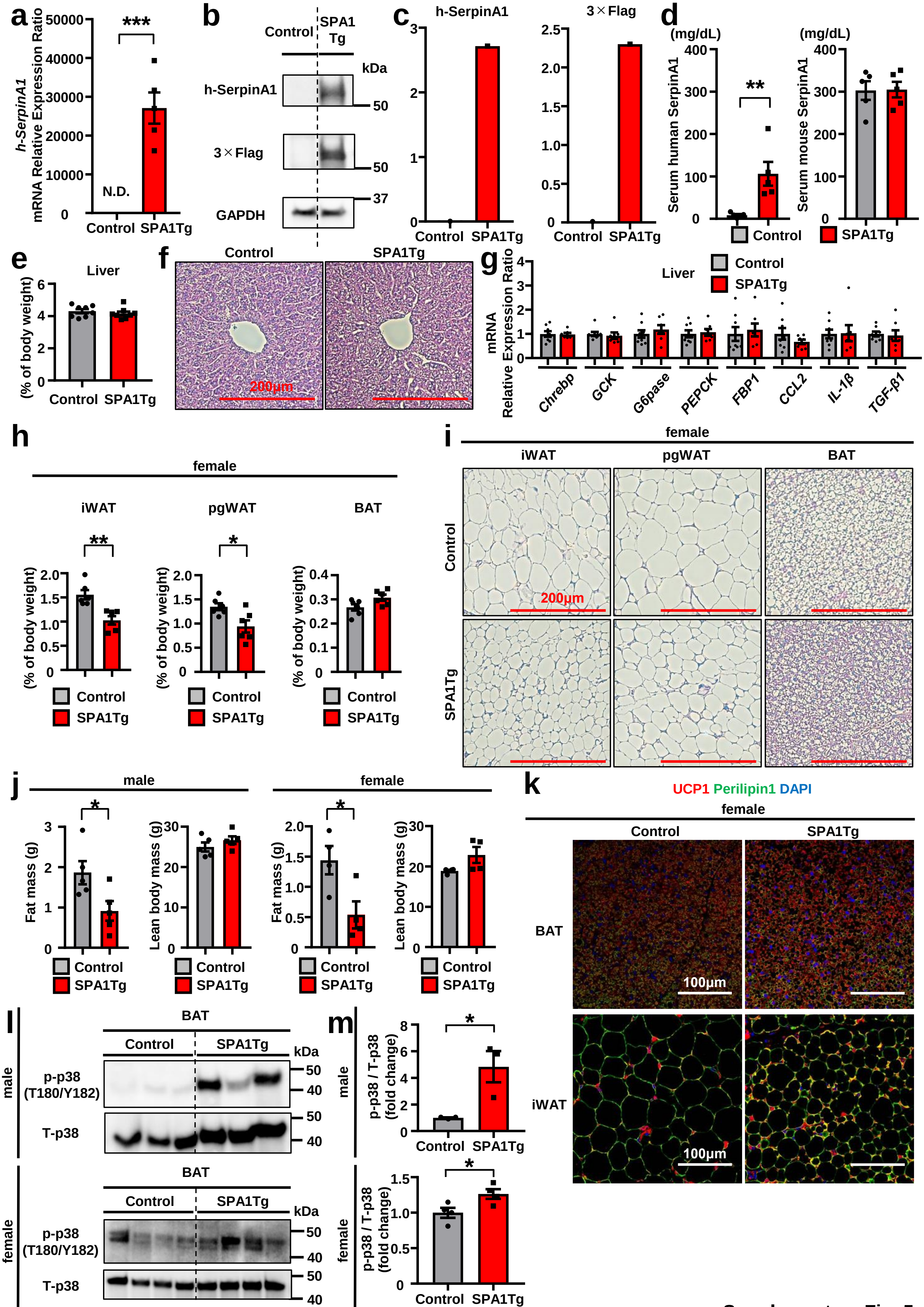

Supplementary Fig. 5

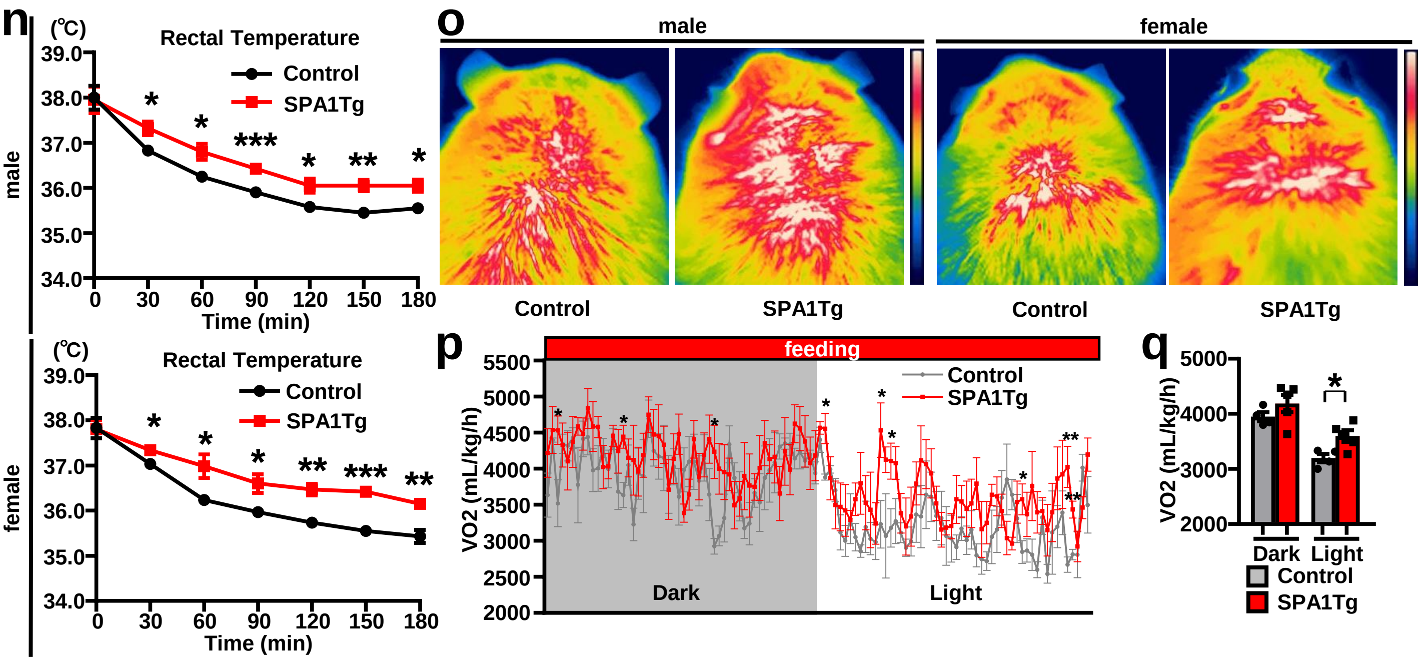

Supplementary Fig. 5 Liver-specific SerpinA1-overexpressing transgenic mice exhibit increased browning and adaptive thermogenesis

All mice in Supplementary Fig. 5 were fed a CD.
(a-g): 12-week-old male control and SPA1Tg mice.
(a): mRNA expression of human *SerpinA1* (*h-SerpinA1*) in livers (Control n = 4, SPA1Tg n = 5, ***p < 0.0001).
(b): The protein levels of h-SerpinA1 and 3×Flag in livers (n = 1).
(c): Quantification of protein levels in (b) (n = 1). Data is presented actual measurements. The data are not statistical evaluations.
(d): ELISA of serum human and mouse SerpinA1 (n = 5, **p = 0.0086).
(e): Liver weight (% of body weight) (n = 8).
(f): HE-stained sections of liver. Scale bars = 200 μm.
(g): mRNA expression in liver (Control n = 8, SPA1Tg n = 7).
(h-i): 12-week-old female control and SPA1Tg mice.
(h): iWAT (**p = 0.0021), pgWAT (*p = 0.0197) and BAT weight (% of body weight) (n = 6).
(i): HE-stained sections of iWAT, pgWAT and BAT. Scale bars = 200 μm.
(j): Fat mass and lean body mass calculated by whole-body micro-CT scan imaging of 12-week-old male (n = 5) and female (n = 4) control and SPA1Tg mice (*p < 0.05).
(k): UCP1- and Perilipin1-immunostained BAT and iWAT sections from 12-week-old female control and SPA1Tg mice.
(l-o): 12-week-old male and female control and SPA1Tg mice.
(l): The protein levels of p-p38 and T-p38 in BAT (male n = 3, female n = 4).
(m): Quantification of protein levels in (i) (male n = 3, female n = 4, *p < 0.05).
(n): Rectal temperatures of male (n = 4) and female (n = 6) exposed to 4 ° C (*p < 0.05, **p < 0.01 and ***p < 0.001).
(o): Thermal images over BAT at 180 minutes of exposure to 4 ° C.
(p): VO_2 of 12-week-old male control and SPA1Tg mice (Control n = 4, SPA1Tg n = 5).
(q): Mean VO_2 in (p) (Control n = 4, SPA1Tg n = 5, *p = 0.0219).
Data are presented as mean ± SEM unless otherwise noted. *P* values were determined using two-tailed Student's t test. Experiments in (f), (i) and (k) were repeated in at least three independent experiments.
Source data are provided as a Source Data file.

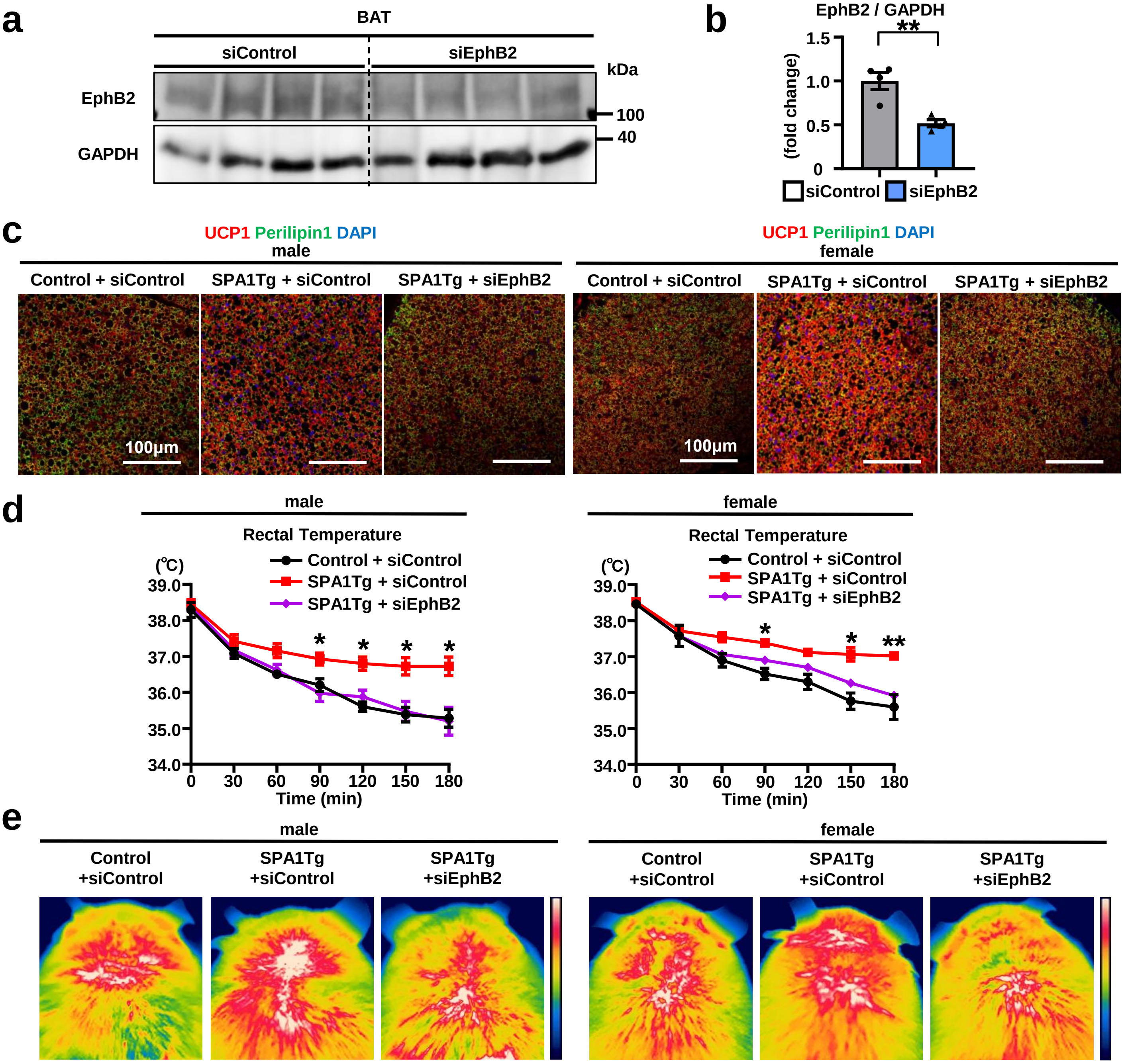

Supplementary Fig. 6 EphB2 plays an important role in heat production of BAT induced by SerpinA1 *in vivo*.

All mice in Supplementary Fig. 6 were fed a CD.
(a): EphB2 protein expression of BAT in male control and SPA1Tg mice directly injected with siControl and siEphB2 into the interscapular BAT (n= 4).
(b): Quantification of protein levels in (a) (n = 4, two-tailed Student's t test, ***p* = 0.0037).
(c-e): Results in 3-month-old male and female control mice directly injected with siControl and SPA1Tg mice directly injected with siControl and siEphB2 into the interscapular BAT.
(c): UCP1- and Perilipin1-immunostained BAT sections. The experiments were repeated at least three times independently.
(d): Rectal temperatures exposed to 4 ° C, 4 days after direct injection into the interscapular BAT (male Control + siControl n = 5, SPA1Tg + siControl n = 4, SPA1Tg + siEphB2 n = 4, female n =5, one-way ANOVA post hoc Bonferroni test, SPA1Tg + siControl vs SPA1Tg + siEphB2, **p* < 0.05 and ***p* < 0.01).
(e): Thermal images showing the temperature over BAT at 180 minutes of exposure to 4 ° C.
Data are presented as mean ± SEM.
Source data are provided as a Source Data file.

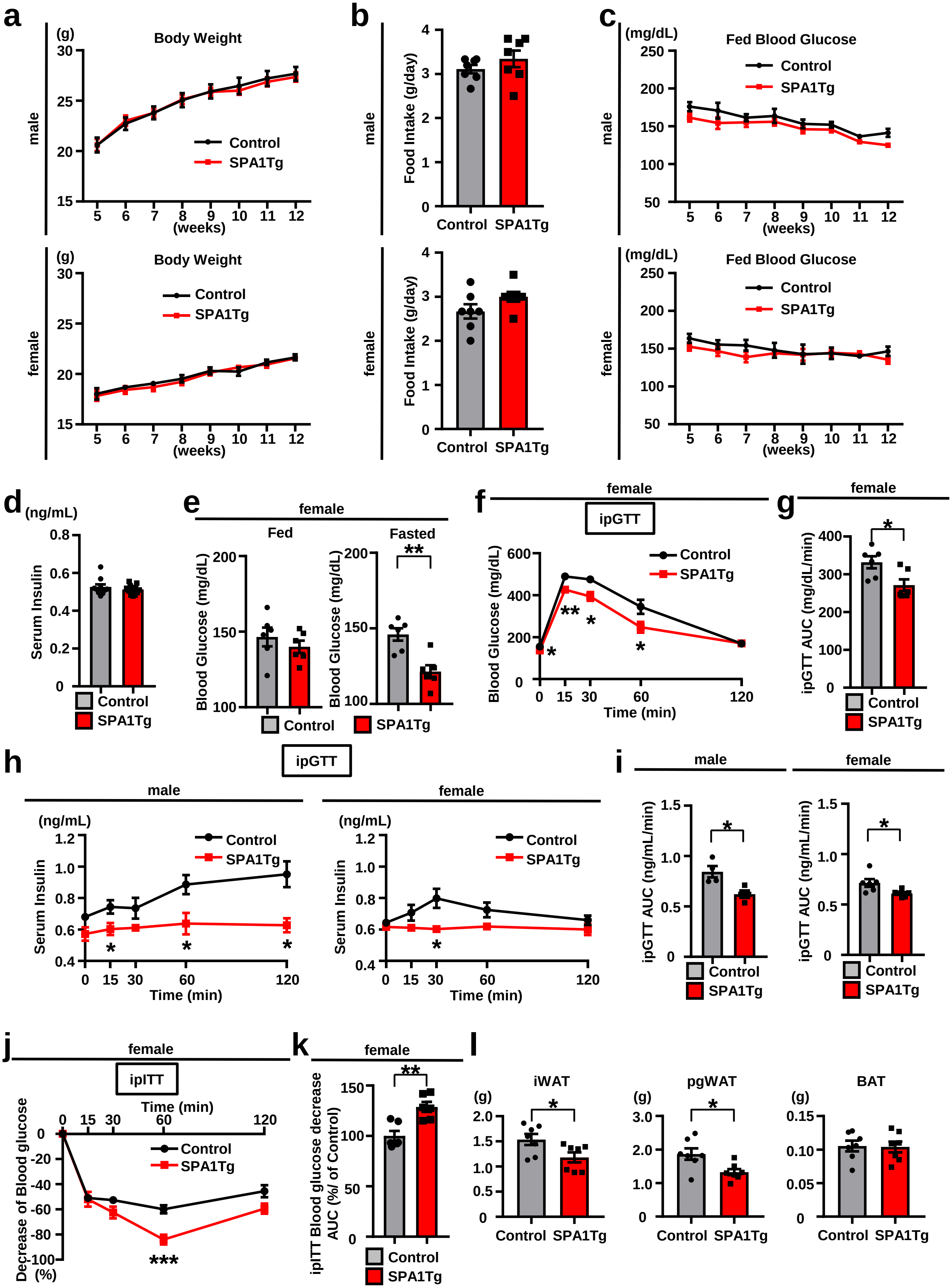

Supplementary Fig. 7

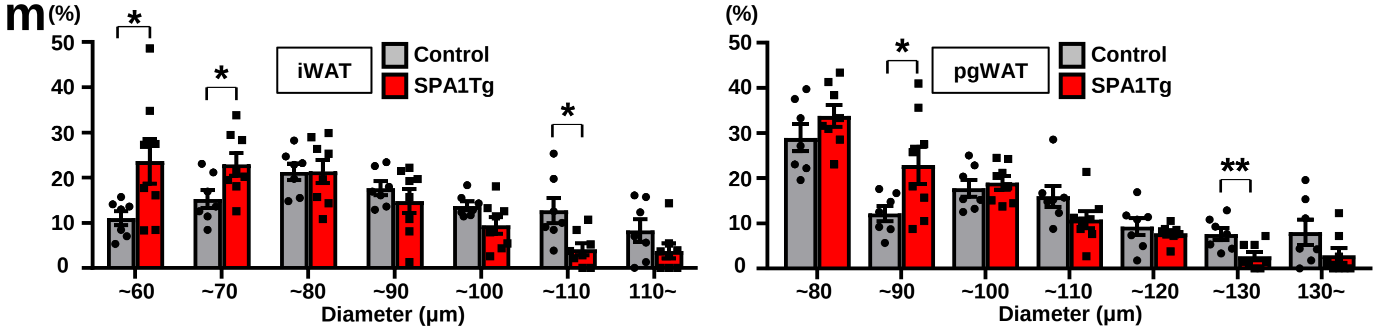

Supplementary Fig. 7 SPA1Tg mice exhibit improved glucose metabolism

(a-c): Mice were fed a CD.
(a): Body weights of 5- to 12-week-old male (Control n = 8, SPA1Tg n = 11) and female (n =6) control and SPA1Tg mice.
(b): Food intake (g/day) of male and female control and SPA1Tg (n = 7).
(c): Fed blood glucose levels of 5- to 12-week-old male (Control n = 8, SPA1Tg n = 11) and female (n = 6) control and SPA1Tg mice.
(d): Fasting serum insulin levels of 12-week-old male control and SPA1Tg mice (Control n = 10, SPA1Tg n = 11).
(e-g): Results in 12-week-old female control and SPA1Tg mice fed a CD.
(e): Fed and fasting blood glucose levels (n = 6, ** $p = 0.0020$).
(f): Results of the ipGTT (n = 6, * $p < 0.05$ and ** $p < 0.01$).
(g): AUC of the ipGTT in (f) (n = 6, * $p = 0.0210$).
(h): Results of plasma insulin levels in the ipGTT for 12-week-old male (n = 4) and female (n = 6) control and SPA1Tg mice (* $p < 0.05$).
(i): AUC of the data in (h) (male n = 4, female n = 6, * $p < 0.05$).
(j): Results of the ipITT for 12-week-old female control and SPA1Tg mice (n = 6, *** $p < 0.001$).
(k): AUC of the ipITT in (j) (n = 6, ** $p = 0.0022$).
(l-m): Results in 17-week-old male HFD-fed control and SPA1Tg mice.
(l): iWAT, pgWAT and BAT mass (g) (n = 7, * $p < 0.05$).
(m): Diameter distributions of iWAT (Control n = 7, SPA1Tg n = 8) and pgWAT (Control n = 7, SPA1Tg n = 8) (* $p < 0.05$ and ** $p < 0.01$).
Data are presented as mean ± SEM. *P* values were determined using two-tailed Student's t test.
Source data are provided as a Source Data file.

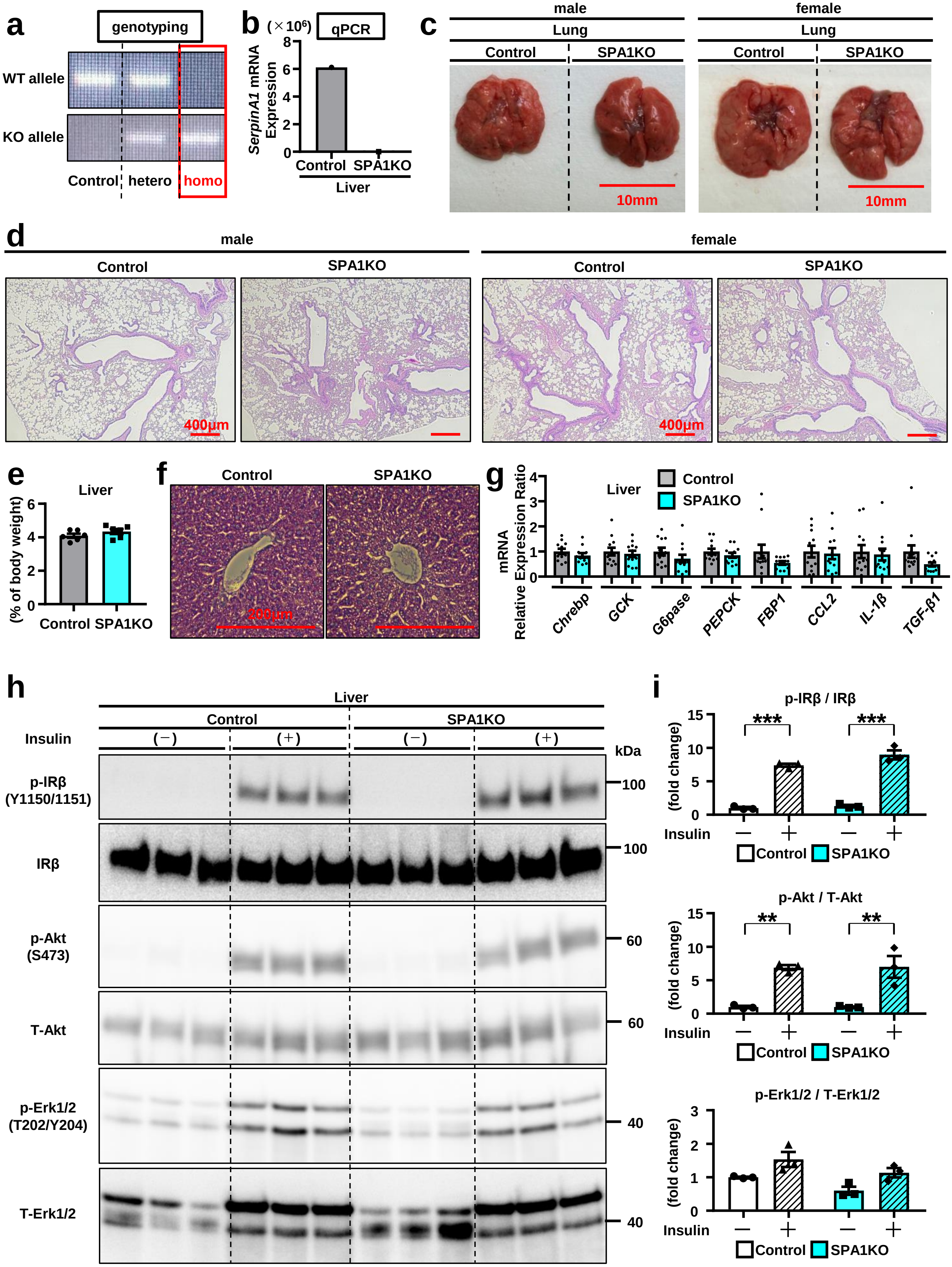

Supplementary Fig. 8

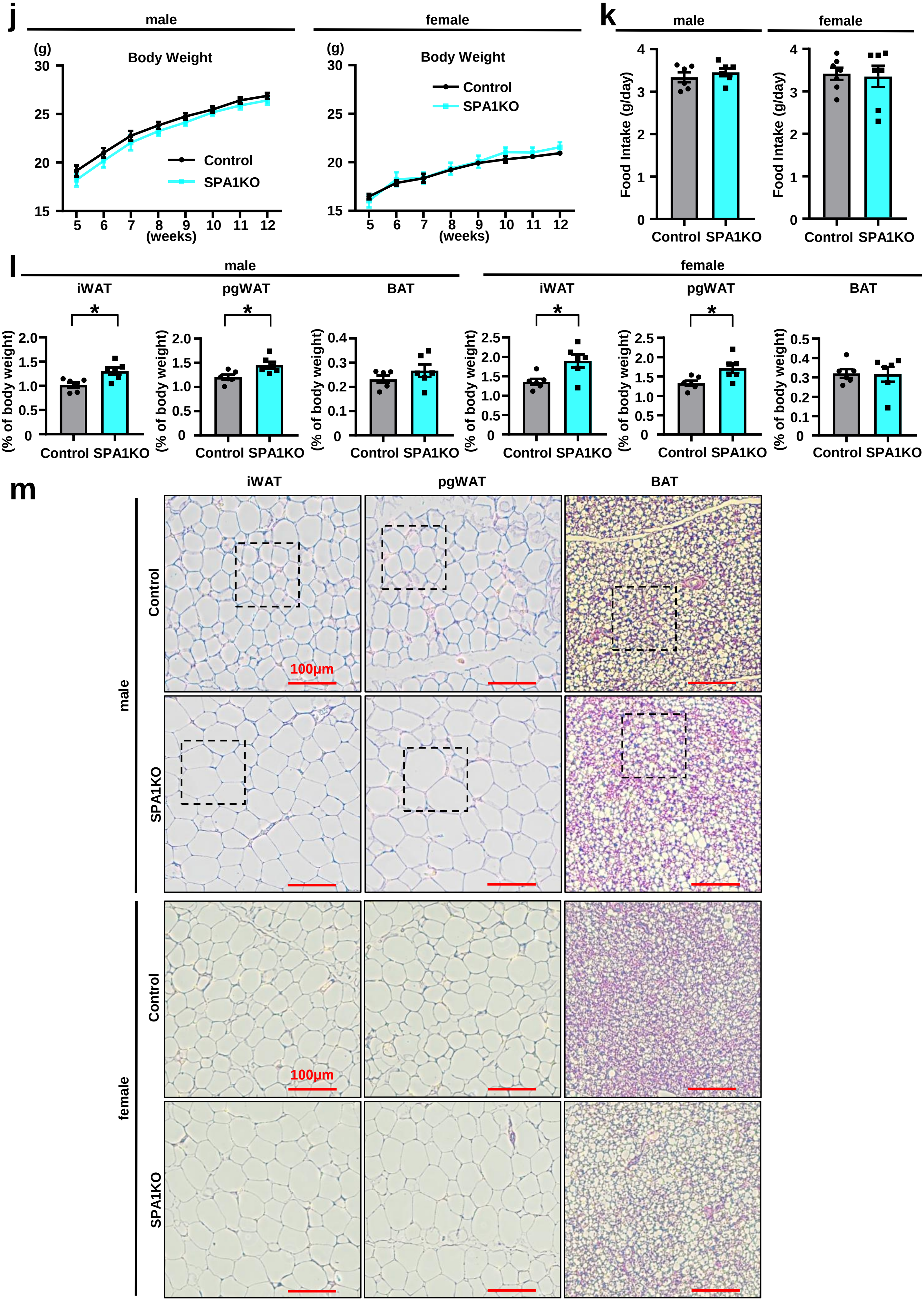

Supplementary Fig. 8

Supplementary Fig. 8 SPA1KO mice exhibit increases in adipose tissue weights and adipocyte diameter

(a-m): Mice were fed a CD.
(a): Identification of mice with SerpinA1＋/＋, SerpinA1＋/－ and SerpinA1－/－ by genotyping.
(b): Relative *SerpinA1* mRNA expression in livers from control and SPA1KO mice (n = 1). Data is presented actual measurements. The data are not statistical evaluations.
(c-d): 12-week-old male and female control and SPA1KO mice.
(c): Representative pictures of lung. Scale bar = 10 mm.
(d): HE-stained sections of lung. Scale bars = 400 μm.
(e-i): 12-week-old male control and SPA1KO mice.
(e): Percentage of tissue weight per body weight of Liver (n = 6).
(f): HE-stained sections of liver. Scale bars = 200 μm.
(g): Relative mRNA expression of genes such as *Chrebp, Gck, G6pase, Pepck, Fbp1, Ccl2, Il-1β* and *Tgf-β1* in liver (n = 12).
(h): Immunoblotting of p-IRβ, p-Akt and p-Erk1/2 in lysates from liver 15 minutes after insulin injection through the inferior vena cava (n = 3).
(i): Quantification of protein levels in (h) (n = 3).
(j): Body weights of 5- to 12-week-old male and female control and SPA1KO mice (male: Control n = 20, SPA1KO n = 14, female: n = 6).
(k): Food intake (g/day) of male (n = 6) and female (n = 7) control and SPA1KO.
(l): Percentage of tissue weight per body weight of iWAT, pgWAT and BAT from 12-week-old male (n = 6) and female (n = 6) control and SPA1KO mice (*$p < 0.05$).
(m): HE-stained sections of iWAT, pgWAT and BAT from 12-week-old male and female control and SPA1KO mice. Scale bars = 100 μm.
Data are presented as mean ± SEM unless otherwise noted. *P* values were determined using two-tailed Student’s t test: (e), (g), (j-l) ; one-way ANOVA post hoc Bonferroni test: (i). Experiments in (d), (f) and (m) were repeated in at least three independent experiments. Source data are provided as a Source Data file.

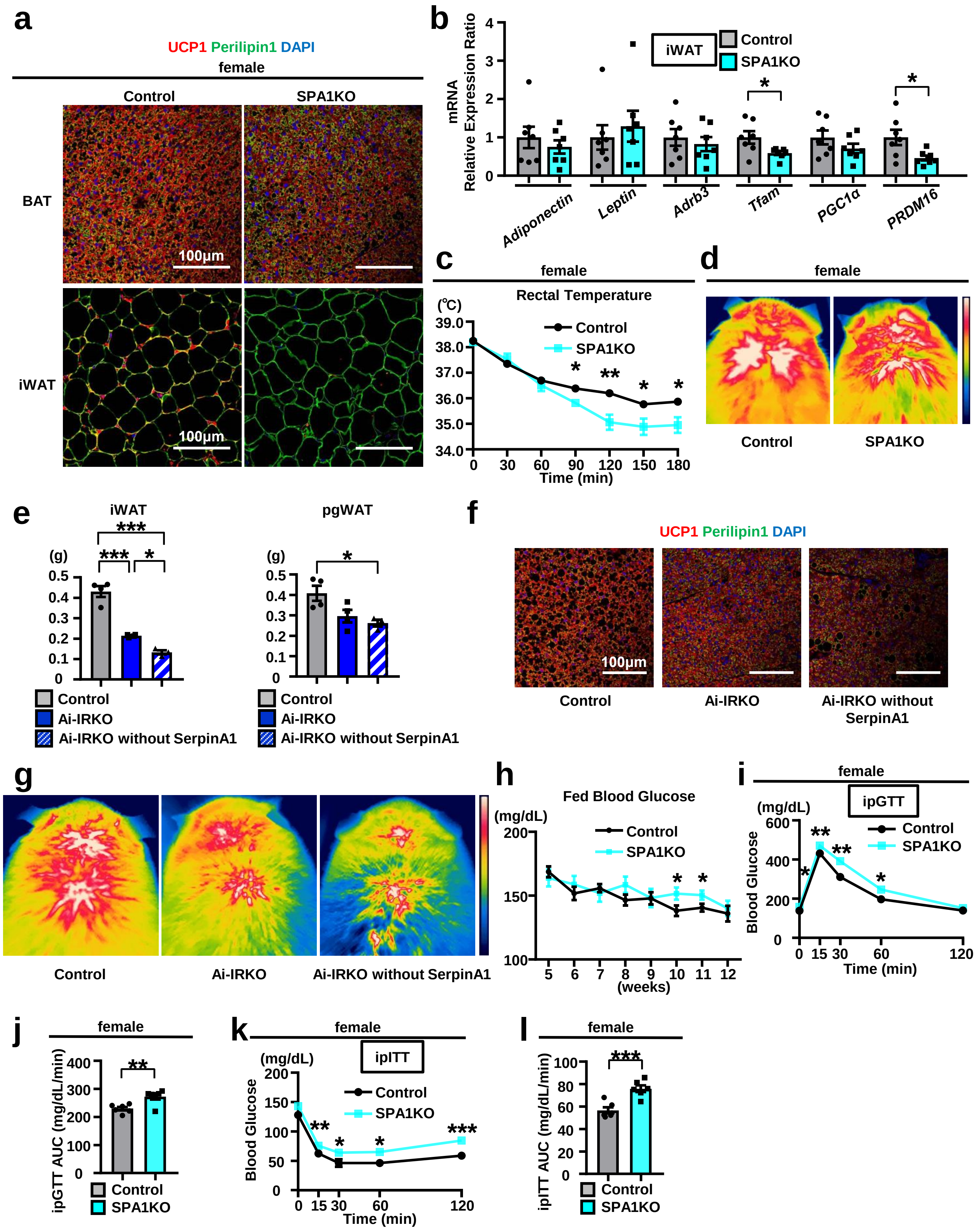

Supplementary Fig. 9

Supplementary Fig. 9 SPA1KO mice exhibit decreased browning and impaired energy expenditure and glucose metabolism

(a-l): Mice were fed a CD.
(a): Representative images of BAT and iWAT sections from 12-week-old female control and SPA1KO mice, immunostained for UCP1 and Perilipin1.
(b): Relative mRNA expression of genes in iWAT from 12-week-old male control and SPA1KO mice (n = 7, *$p < 0.05$).
(c): Rectal temperatures of 12-week-old female control and SPA1KO mice exposed to 4 ° C (n = 6, *$p < 0.05$ and **$p < 0.01$).
(d): Thermal images showing the surface temperature over BAT in 12-week-old female control and SPA1KO mice at 120 minutes of exposure to 4 ° C.
(e): Tissue weights (g) of iWAT and pgWAT from male control, Ai-IRKO and Ai-IRKO without SerpinA1 mice after tamoxifen injection (Control n = 4, Ai-IRKO n = 4, Ai-IRKO without SerpinA1 n = 3, *$p < 0.05$ and ***$p < 0.001$).
(f): UCP1- and Perilipin1-immunostained BAT sections from male control, Ai-IRKO and Ai-IRKO without SerpinA1 mice after tamoxifen injection.
(g): Thermal images showing the temperature over BAT in male control, Ai-IRKO and Ai-IRKO without SerpinA1 mice after tamoxifen injection at 180 minutes of exposure to 4 ° C.
(h): Fed Blood glucose levels of 5- to 12-week-old male control and SPA1KO mice (Control n = 20, SPA1KO n = 14, *$p < 0.05$).
(i): Results of the ipGTT for 12-week-old female control and SPA1KO mice (n = 6, *$p < 0.05$ and **$p < 0.01$).
(j): AUC of the ipGTT in (i) (n = 6, **$p = 0.0059$).
(k): Results of the ipITT for 12-week-old female control and SPA1KO mice (n = 6, *$p < 0.05$, **$p < 0.01$ and ***$p < 0.001$).
(l): AUC of the ipITT in (k) (n = 6, ***$p = 0.0008$).
Data are presented as mean ± SEM unless otherwise noted. *P* values were determined using two-tailed Student’s t test: (b-c), (h-l); one-way ANOVA post hoc Bonferroni test: (e). Experiments in (a) and (f) were repeated in at least three independent experiments.
Source data are provided as a Source Data file.

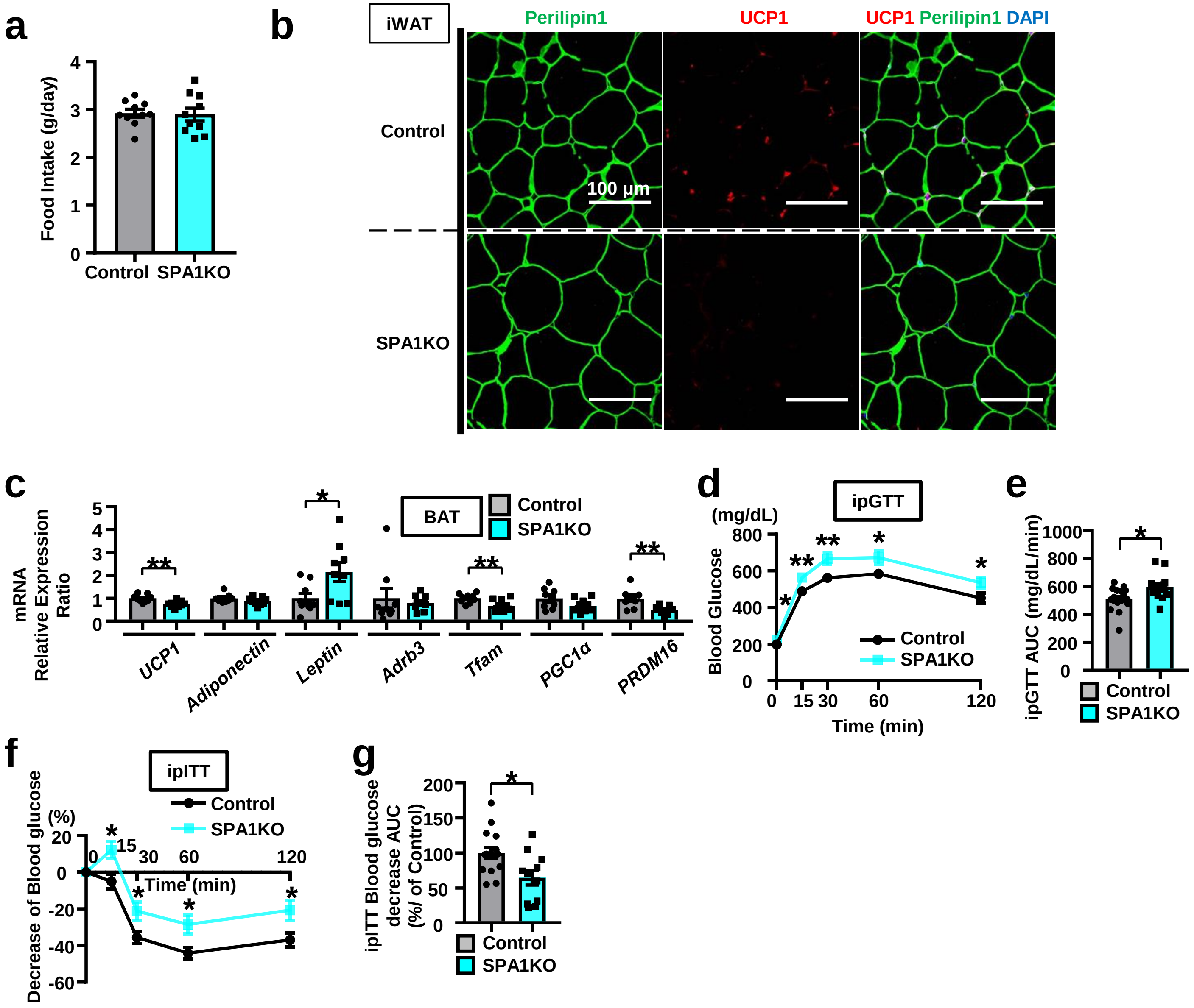

Supplementary Fig. 10 HFD-fed SPA1KO mice exhibit impaired glucose metabolism

(a-g): Results in 17-week-old male HFD-fed control and SPA1KO mice.
(a): Food intake (g/day) (n = 10).
(b): UCP1- and Perilipin1-immunostained iWAT sections. Scale bars = 100 μm. The experiments were repeated at least three times independently.
(c): Relative mRNA expression of genes in BAT (n = 9, *p < 0.05 and **p < 0.01).
(d): Results of the ipGTT (Control n = 18, SPA1KO n = 13, *p < 0.05 and **p < 0.01).
(e): AUC of the ipGTT in (d) (Control n = 18, SPA1KO n = 13, *p = 0.0104).
(f): Results of the ipITT (Control n = 15, SPA1KO n = 11, *p < 0.05).
(g): AUC of the ipITT in (f) (Control n = 15, SPA1KO n = 11, *p = 0.0129).
Data are presented as mean ± SEM. P values were determined using two-tailed Student's t test.
Source data are provided as a Source Data file.

Supplementary Fig. 11 Uncut blots.

The red sections indicate blot results shown in Supplementary Figure.

Supplementary Fig. 2b

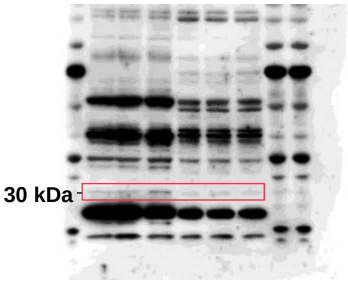

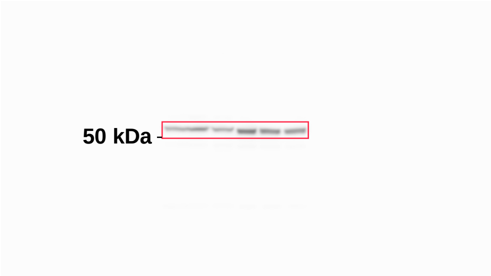

Supplementary Fig. 2e

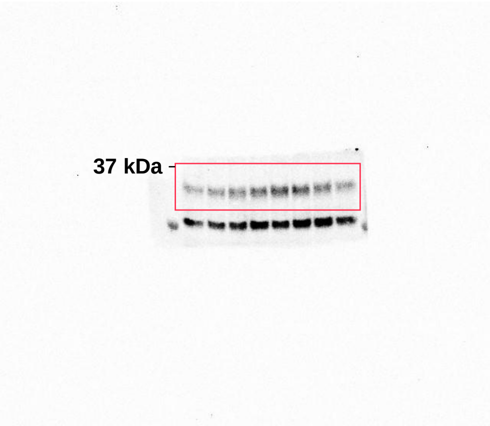

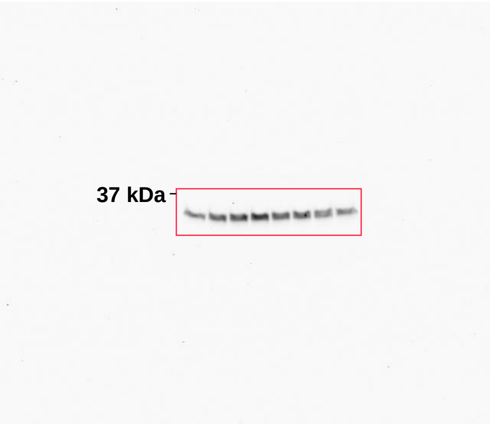

Supplementary Fig. 3a

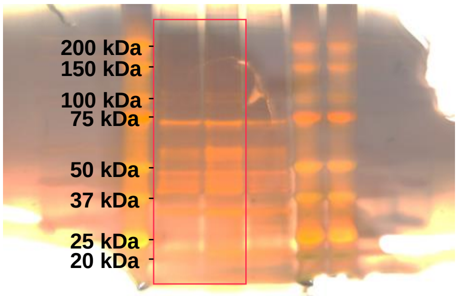

Supplementary Fig. 3b

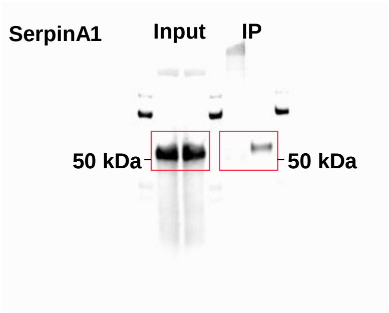

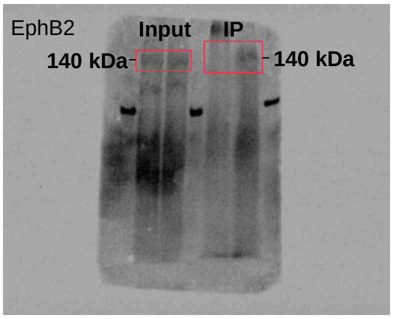

Supplementary Fig. 3c

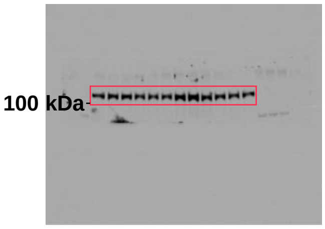

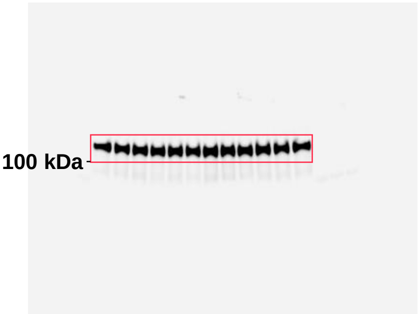

Supplementary Fig. 4a

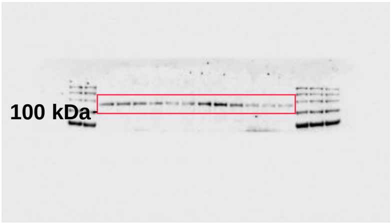

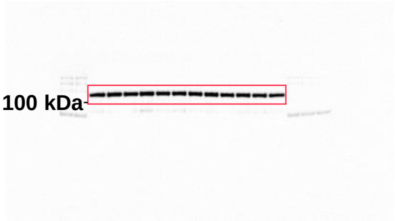

Supplementary Fig. 5b

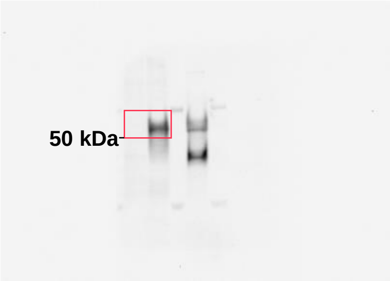

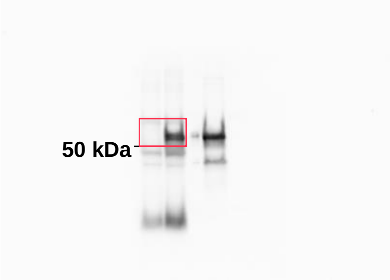

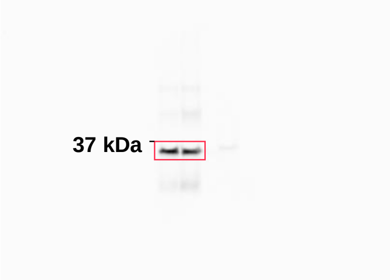

Supplementary Fig. 5l

male

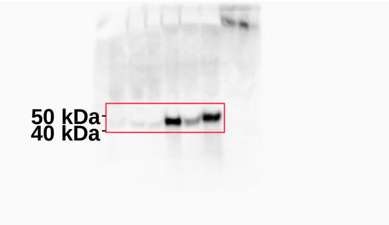

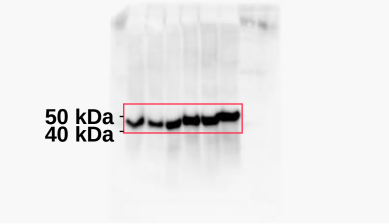

female

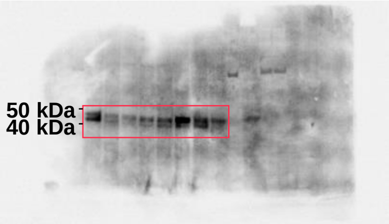

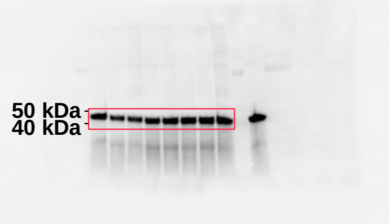

Supplementary Fig. 6a

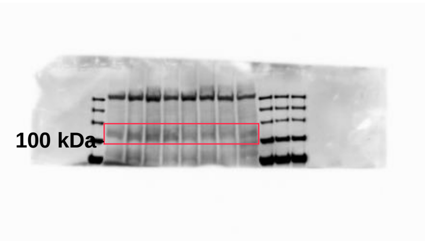

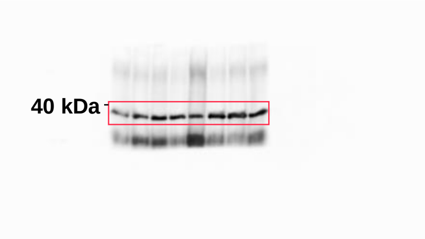

Supplementary Fig. 8h

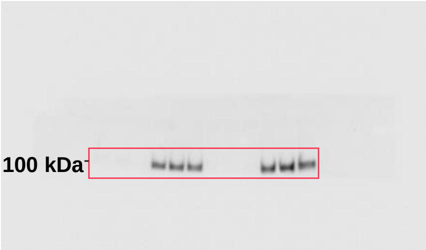

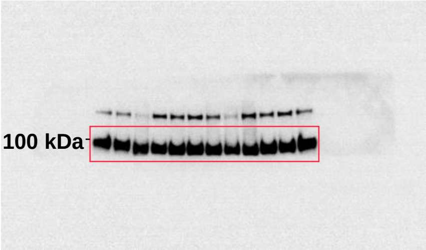

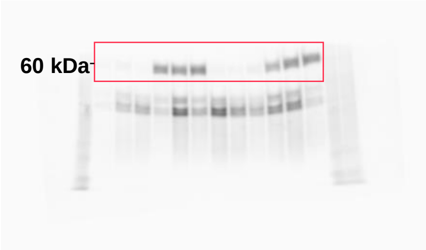

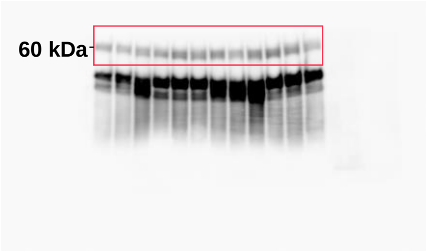

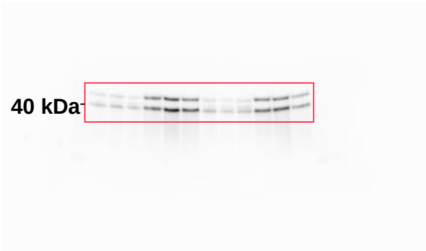

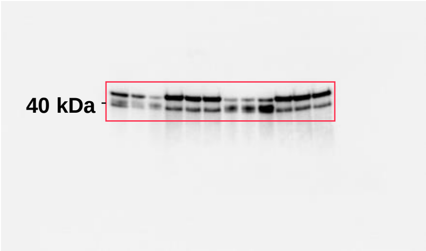

Supplementary Table 1. Quantitative Real-Time PCR primer sequences (mouse)

Gene	Direction	Sequence
TBP	Forward	ACCCTTCACCAATGACTCCTATG
TBP	Reverse	TGACTGCAGCAAATCGCTTGG
SerpinA1(a-e)	Forward	GATGGGAAGATGCAGCATC
SerpinA1(a-e)	Reverse	TCCAGAGATGGACAGTCTG
SerpinA1a	Forward	GATGAGACAGGAACAGAAGCT
SerpinA1a	Reverse	CATAGGAACCATTTGTAAGA
SerpinA1b	Forward	GATGAGACAGGAACAGAAGCT
SerpinA1b	Reverse	CATAGGAACGGCTTCAAAGA
SerpinA1c	Forward	GATGAGACAGGAACAGAAGCT
SerpinA1c	Reverse	AACGGCTAGTAAGACTGTAG
SerpinA1d	Forward	GATGAGACAGGAACAGAAGCT
SerpinA1d	Reverse	ATAAGTAGCGACTTGTAAGA
SerpinA1e	Forward	GATGAGACAGGAACAGAAGCT
SerpinA1e	Reverse	CAAAAAACCGCCTTGTAAGA
Adiponectin	Forward	GATGGCACTCCTGGAGAGAA
Adiponectin	Reverse	GCTTCTCCAGGCTCTCCTTT
Leptin	Forward	GGGCTTCACCCCATTCTGA
Leptin	Reverse	TGGCTATCTGCACATTTTG
FAS	Forward	GAGGACACTCAAGTGGCTGA
FAS	Reverse	GTGAGGTTGCTGTCGTCTGT
ATGL	Forward	ACTGTGGCCTCATTCCTCCT
ATGL	Reverse	AACTGGATGCTGGTGTTGGT
Glut4	Forward	ATCTTGATGACCGTGGCTCT
Glut4	Reverse	CTCAAAGAAGGCCACAAAGC
PPARγ	Forward	TGTTATGGGTGAAACTCTGGG
PPARγ	Reverse	AGAGCTGATTCCGAAGTTGG
AP2	Forward	GATGCCTTTGTGGGAACCT
AP2	Reverse	CTGTCGTCTGCGGTGATTT
CEBPα	Forward	CAAGAACAGCAACGAGTACCG
CEBPα	Reverse	GTCACTGGTCAACTCCAGCAC

Gene	Direction	Sequence
Adrb3	Forward	GCTGACTTGGTAGTGGGACTC
Adrb3	Reverse	TAGAAGGAGACGGAGGAGGAG
Tfam	Forward	AGTTCCCACGCTGGTAGTGT
Tfam	Reverse	GCGCACATCTCGACCC
Elovl3	Forward	GGACTTAAGGCCCTTTTTGG
Elovl3	Reverse	TTCCGCGTTCTCATGTAGGT
Cidea	Forward	ATCACAACTGGCCTGGTTACG
Cidea	Reverse	TACTACCCGGTGTCCATTTCT
PGC1α	Forward	CCCTGCCATTGTTAAGACC
PGC1α	Reverse	TGCTGCTGTTCCTGTTTTC
PRDM16	Forward	CAGCACGGTGAAGCCATTC
PRDM16	Reverse	GCGTGCATCCGCTTGTG
UCP1	Forward	ACTGCCACACCTCCAGTCATT
UCP1	Reverse	CTTTGCCTCACTCAGGATTGG
Chrebp	Forward	CTGGGGACCTAAACAGGAGC
Chrebp	Reverse	GAAGCCACCCTATAGCTCCC
GCK	Forward	CAACTGGACCAAGGGCTTCAA
GCK	Reverse	TGTGGCCACCGTGTCATTC
G6Pase	Forward	GCCAGAATGGGTCCACCTTG
G6Pase	Reverse	TGCAGGAGGACCAAGGAAGC
PEPCK	Forward	TTTGCCATGCGACCCTTCTT
PEPCK	Reverse	CTTCAATCCGCCCGAACATC
FBP1	Forward	CCATCATAATCGAACCTGAG
FBP1	Reverse	CTTCTCAGAAGGCTCATCAG
CCL2	Forward	TTAAAAACCTGGATCGGAACCAA
CCL2	Reverse	GCATTAGCTTCAGATTTACGGGT
IL-1β	Forward	GCAACTGTTCCTGAACTCAACT
IL-1β	Reverse	ATCTTTTGGGGTCCGTCAACT
TGF-β1	Forward	AAGTTGGCATGGTAGCCCTT
TGF-β1	Reverse	GCCCTGGATACCAACTATTGC

Supplementary Table 2. Quantitative Real-Time PCR primer sequences (human)

Gene	Direction	Sequence
UCP1	Forward	ACCGCAGGGAAAGAAACAGC
UCP1	Reverse	TCAGATTGGGAGTAGTCCCT
Adiponectin	Forward	TTCACCGATGTCTCCCTTAGG
Adiponectin	Reverse	GGCATGACCAGGAAACCAC
Leptin	Forward	TCTATGTCCAAGCTGTGC
Leptin	Reverse	TTGGAGGAGACTGACTGC
PGC1α	Forward	AGTGGTGCAGTGACCAATCA
PGC1α	Reverse	CTGCTAGCAAGTTTGCCTCA
AP2	Forward	ACTGGGCCAGGAATTTGACGAAGT
AP2	Reverse	TCTCGTGGAAGTGACGCCTTTCAT
FAS	Forward	GCATCTGGACCCTCCTACCT
FAS	Reverse	TCCTCAATTCCAATCCCTTG
Dio2	Forward	CCTCCTCGATGCCTACAAAC
Dio2	Reverse	GCTGGCAAAGTCAAGAAGGT
Pat2	Forward	CCTGCCACTGTATGCACATC
Pat2	Reverse	TAGTCCATGCATCACCGTGT
CD137	Forward	AGCTGTTACAACATAGTAGCCAC
CD137	Reverse	TCCTGCAATGATCTTGTCCTCT
CD40	Forward	GAGTTCACTGAAACGGAATGCC
CD40	Reverse	GTCTCTCTGTTCCAGGTGTCT
CITED1	Forward	CAACCTTGCGGTGAAAGATCG
CITED1	Reverse	GGAGAGCCTATTGGAGATCCC
Sp100	Forward	TCCCCATCTCATGCTGGTACA
Sp100	Reverse	TGGCTTCCTAGCGAATCATCTT
SerpinA1	Forward	ATGATCTGAAGAGCGTCCTG
SerpinA1	Reverse	AGCTTCAGTCCCTTTCTCGT
