# Supplementary material for: Hepatic SerpinA1 improves energy and glucose metabolism through regulation of preadipocyte proliferation and UCP1 expression
Source: Nat Commun. 2024 Nov 12;15:9585. doi: 10.1038/s41467-024-53835-9 (PMC11557585; doi:10.1038/s41467-024-53835-9)
Supplement: Supplementary file 2 — Reporting Summary [file 41467_2024_53835_MOESM2_ESM.pdf]

nature portfolio

Corresponding author(s): Masaji Sakaguchi

Last updated by author(s): Oct 3, 2024

Reporting Summary

Nature Portfolio wishes to improve the reproducibility of the work that we publish. This form provides structure for consistency and transparency in reporting. For further information on Nature Portfolio policies, see our Editorial Policies and the Editorial Policy Checklist.

Statistics

For all statistical analyses, confirm that the following items are present in the figure legend, table legend, main text, or Methods section.

n/a	Confirmed	
☐	☒	The exact sample size (*n*) for each experimental group/condition, given as a discrete number and unit of measurement
☐	☒	A statement on whether measurements were taken from distinct samples or whether the same sample was measured repeatedly
☐	☒	The statistical test(s) used AND whether they are one- or two-sided<br>*Only common tests should be described solely by name; describe more complex techniques in the Methods section.*
☒	☐	A description of all covariates tested
☐	☒	A description of any assumptions or corrections, such as tests of normality and adjustment for multiple comparisons
☐	☒	A full description of the statistical parameters including central tendency (e.g. means) or other basic estimates (e.g. regression coefficient) AND variation (e.g. standard deviation) or associated estimates of uncertainty (e.g. confidence intervals)
☐	☒	For null hypothesis testing, the test statistic (e.g. *F*, *t*, *r*) with confidence intervals, effect sizes, degrees of freedom and *P* value noted<br>*Give P values as exact values whenever suitable.*
☒	☐	For Bayesian analysis, information on the choice of priors and Markov chain Monte Carlo settings
☒	☐	For hierarchical and complex designs, identification of the appropriate level for tests and full reporting of outcomes
☒	☐	Estimates of effect sizes (e.g. Cohen's *d*, Pearson's *r*), indicating how they were calculated

Our web collection on statistics for biologists contains articles on many of the points above.

Software and code

Policy information about availability of computer code

Data collection	Cooled CCD Camera System Light-Capture II (ATTO), WSE-6170 LuminoGraph I CMOS (ATTO) and ChemiDoc™ Touch Gel Imaging System (BIO-RAD) was used to acquire western blot images.<br>CS Analyzer version 2.0 (ATTO) was used to export and digitize western blot images.<br>Seahorse XFe24 Flux Analyzer (Agilent Technologies).<br>Confocal Laser Scanning Microscope (OLYMPUS, FV3000).<br>All-in-One Fluorescence Microscope (Keyence, BZ-9000).<br>FLIR E53 Advanced Thermal Imaging Infrared Camera (Teledyne FLIR).<br>Implantable Temperature Transponders (BioMedic Data Systems, Inc., IPTT-300).<br>In-vivo Micro-CT scanners (ALOKA, LaTheta LCT-100).
Data analysis	Excel version 2407.<br>Graph pad prism version 7.<br>File image J 1.52a.<br>BZ-II Dynamic Cell Count Analyzer (Keyence, BZ-H1CE).<br>Adiposoft image analysis software version 1.16.

For manuscripts utilizing custom algorithms or software that are central to the research but not yet described in published literature, software must be made available to editors and reviewers. We strongly encourage code deposition in a community repository (e.g. GitHub). See the Nature Portfolio guidelines for submitting code & software for further information.

Data

Policy information about availability of data

All manuscripts must include a data availability statement. This statement should provide the following information, where applicable:

- Accession codes, unique identifiers, or web links for publicly available datasets
- A description of any restrictions on data availability
- For clinical datasets or third party data, please ensure that the statement adheres to our policy

The authors declare that all data generated in this study is provided in the Supplementary Information/Source Data file. Source Data are provided with this paper. The raw mass spectrometry data (related Fig.1b) have been deposited in the ProteomeXchange Consortium via the PRIDE partner repository under accession code PXD047144. The data are also available through the MassIVE repository (massive.ucsd.edu) with the dataset accession number MSV000093454. The raw mass spectrometry proteomics data (related Fig.3i) have been deposited in the ProteomeXchange Consortium via the PRIDE partner repository under accession code PXD056002.

Research involving human participants, their data, or biological material

Policy information about studies with human participants or human data. See also policy information about sex, gender (identity/presentation), and sexual orientation and race, ethnicity and racism.

Reporting on sex and gender	This study did not involve human research participants.
Reporting on race, ethnicity, or other socially relevant groupings	This study did not involve human research participants.
Population characteristics	This study did not involve human research participants.
Recruitment	This study did not involve human research participants.
Ethics oversight	This study did not involve human research participants.

Note that full information on the approval of the study protocol must also be provided in the manuscript.

Field-specific reporting

Please select the one below that is the best fit for your research. If you are not sure, read the appropriate sections before making your selection.

☒ Life sciences ☐ Behavioural & social sciences ☐ Ecological, evolutionary & environmental sciences

For a reference copy of the document with all sections, see nature.com/documents/nr-reporting-summary-flat.pdf

Life sciences study design

All studies must disclose on these points even when the disclosure is negative.

Sample size	No statistical method was used to predetermine sample size. The cell-based experiments and the animal experiments, n=3-20 per group per experiment was chosen based on our previous experience with similar in vivo and in vitro experiments (PMID: 30952843, PMID:28065828 and PMID: 36241662).
Data exclusions	No data were excluded from the analysis.
Replication	The number of biological independent replicates indicated in the figure legends.
Randomization	The allocations of fluorescence images and H&E image analysis used this study were randomized by performing analysis at random positions. For in vivo studies, allocation of animals was randomly assigned to different groups, and all mice were matched for age and sex within the same experiment.
Blinding	Although the primary investigators were not blinded for most of the studies, the investigators performing some of the imaging analysis and the sample analyses (such as measurement of blood parameters) were notinformed ofthe expected results of the experiment before or during the analysis. Additionally, proteomics were performed blinded.

Reporting for specific materials, systems and methods

We require information from authors about some types of materials, experimental systems and methods used in many studies. Here, indicate whether each material, system or method listed is relevant to your study. If you are not sure if a list item applies to your research, read the appropriate section before selecting a response.

Materials & experimental systems

n/a	Involved in the study
☐	☒ Antibodies
☐	☒ Eukaryotic cell lines
☒	☐ Palaeontology and archaeology
☐	☒ Animals and other organisms
☒	☐ Clinical data
☒	☐ Dual use research of concern
☒	☐ Plants

Methods

n/a	Involved in the study
☒	☐ ChIP-seq
☒	☐ Flow cytometry
☒	☐ MRI-based neuroimaging

Antibodies

Antibodies used

phospho-IR/IGF1R, 19H7, Cell Signaling Technology, (#3024, 1:1000)
IRβ, 4B8, Cell Signaling Technology, (#3025, 1:1000)
phospho-p44/42 MAPK (Erk1/2) (Thr202/Tyr204), Cell Signaling Technology, (#9101, 1:1000)
p44/42 MAPK (Erk1/2), Cell Signaling Technology, (#9102, 1:1000)
phospho-Akt (S473), Cell Signaling Technology, (#9271, 1:1000)
Akt, 11E7, Cell Signaling Technology, (#4685, 1:1000)
phospho-p38 MAPK, D3F9, (Thr180/Tyr182), Cell Signaling Technology, (#4511, 1:1000)
p38 MAPK, Cell Signaling Technology, (#9212, 1:1000)
phospho-FAK (Tyr397), Cell Signaling Technology, (#3283, 1:1000)
FAK, Cell Signaling Technology, (#3285, 1:1000)
GAPDH, D4C6R, Cell Signaling Technology, (#97166, 1:1000)
β-Actin, 13ES, Cell Signaling Technology, (#4970, 1:1000)
α-Tubulin, Cell Signaling Technology, (#2144, 1:1000)
Flag, Sigma, (F3040, 1:1000)
UCP1, Abcam, (ab10983,1:100-1:1000)
Perilipin1, Abcam, (ab61682, 1:100)
SerpinA1 (alpha 1 Antitrypsin), Abcam, (ab166610, 1:1000)
SerpinA1 (alpha 1 Antitrypsin), Abcam, (ab205152, 1:1000)
SerpinA1 (alpha 1 Antitrypsin), Proteintech Group, Inc., (16382-1-AP, 4.0 μg for 3.0 mg of total protein lysate)
EphB2, R&D Systems, Inc., (AF467, 1:1000)
Anti-rabbit IgG, HRP-linked Antibody, Cell Signaling Technology, (#7074, 1:1000)
Anti-mouse IgG, HRP-linked Antibody, Cell Signaling Technology, (#7076, 1:1000)
Anti-rat IgG, HRP-linked Antibody, Cell Signaling Technology, (#7076, 1:1000)
mouse anti-goat IgG-HRP, Santa Cruz, (sc-2354, 1:1000)
Donkey anti-Rabbit IgG (H+L) Highly Cross-Adsorbed Secondary Antibody, Alexa Fluor Plus 594, Invitrogen, (A32754, 1:500)
Donkey anti-Goat IgG (H+L) Highly Cross-Adsorbed Secondary Antibody, Alexa Fluor Plus 488, Invitrogen, (A32814, 1:500)

Validation

The primary antibodies for phospho-IR/IGF1R, 19H7 (#3024, https://www.cellsignal.jp/products/primary-antibodies/phospho-igf-i-receptor-b-tyr1135-1136-insulin-receptor-b-tyr1150-1151-19h7-rabbit-mab/3024), IRβ, 4B8 (#3025, https://www.cellsignal.jp/products/primary-antibodies/insulin-receptor-b-4b8-rabbit-mab/3025), phospho-p44/42 MAPK (Erk1/2) (Thr202/Tyr204) (#9101, https://www.cellsignal.jp/products/primary-antibodies/phospho-p44-42-mapk-erk1-2-thr202-tyr204-antibody/9101), p44/42 MAPK (Erk1/2) (#9102, https://www.cellsignal.jp/products/primary-antibodies/p44-42-mapk-erk1-2-antibody/9102), phospho-Akt (S473) (#9271, https://www.cellsignal.jp/products/primary-antibodies/phospho-akt-ser473-antibody/9271), Akt, 11E7 (#4685, https://www.cellsignal.jp/products/primary-antibodies/akt-pan-11e7-rabbit-mab/4685), phospho-p38 MAPK, D3F9 (Thr180/Tyr182) (#4511, https://www.cellsignal.jp/products/primary-antibodies/phospho-p38-mapk-thr180-tyr182-d3f9-xp-rabbit-mab/4511), p38 MAPK (#9212, https://www.cellsignal.jp/products/primary-antibodies/p38-mapk-antibody/9212), phospho-FAK (Tyr397) (#3283, https://www.cellsignal.jp/products/primary-antibodies/phospho-fak-tyr397-antibody/3283), FAK (#3285, https://www.cellsignal.jp/products/primary-antibodies/fak-antibody/3285), GAPDH, D4C6R (#97166, https://www.cellsignal.jp/products/primary-antibodies/gapdh-d4c6r-mouse-mab/97166), β-Actin, 13E5 (#4970, https://www.cellsignal.jp/products/primary-antibodies/b-actin-13e5-rabbit-mab/4970) and α-Tubulin (#2144, https://www.cellsignal.jp/products/primary-antibodies/a-tubulin-antibody/2144) were validated for the western blotting for both human and mouse samples on the website of the Cell Signaling Technology. SerpinA1 (alpha 1 Antitrypsin) (ab166610, https://www.abcam.co.jp/products/primary-antibodies/alpha-1-antitrypsin-antibody-epr9090-ab166610.html) antibody was validated for the western blotting for the human sample on the website of the company. SerpinA1 (alpha 1 Antitrypsin) (ab205152) antibody was validated for the western blotting for the murine sample. EphB2 (AF467, https://www.rndsystems.com/products/human-mouse-ephb2-antibody_af467) antibody was validated for the western blotting for both human and murine sample on the website of the company. Flag (F3040, https://www.sigmaaldrich.com/JP/ja/product/sigma/f3040) and UCP1 (ab10983, https://www.abcam.co.jp/products/primary-antibodies/ucp1-antibody-ab10983.html) antibodies were validated for the western blotting on the website of the company. UCP1 (ab10983, https://www.abcam.co.jp/products/primary-antibodies/ucp1-antibody-ab10983.html), Perilipin1 (ab61682, www.abcam.eo.jp/perilipin-1-antibody-ab61682.html) antibodies were validated for IHC studies of the mouse samples on the websites of the company. SerpinA1 (alpha 1 Antitrypsin) (16382-1-AP, https://www.ptglab.co.jp/products/SERPINA1-Antibody-16382-1-AP.htm) antibody was validated for IP studies of the mouse samples on the websites of the company.

Eukaryotic cell lines

Policy information about cell lines and Sex and Gender in Research

Cell line source(s)	Immortalized mouse brown preadipocytes (WT-1) were derived from the stromal vascular fraction (SVF) of interscapular brown adipose tissue of newborn mice. The SVF cells were immortalized by SV40 T overexpression as described in PMID 14966273 and PMID: 18719589. A41 hWAT-SVF cells were human white preadipocytes collected from a human male subject. The SVF cells were immortalized by hTert overexpression as described in PMID: 26076036. Adeno-X™ 293T cells (Takara, 632271). Lenti-X™ 293T cells (Takara, 632180).
Authentication	The immortalized mouse brown preadipocytes (WT-1) have been deposited to Millipore Sigma and were authenticated by Millipore Sigma (#SCC255).<br>The immortalized human white preadipocytes (A41 hWAT-SVF) have been deposited to ATCC and were authenticated by ATCC (#CRL-3386).
Mycoplasma contamination	Cell lines were screened for mycoplasma and they were negative for mycoplasma.
Commonly misidentified lines (See ICLAC register)	No commonly misidentified lines were used.

Animals and other research organisms

Policy information about studies involving animals; ARRIVE guidelines recommended for reporting animal research, and Sex and Gender in Research

Laboratory animals	Mice were housed at 20–22 °C on a 12 h light/dark cycle with average 50% Humidity and fed either a CD (#Rodent Diet CE-2, CLEA, Tokyo, Japan) or HFD (60% calories from fat; #HFD32, CLEA, Tokyo, Japan) in the animal facility at Kumamoto University, Japan. All experiments with research animals were performed in accordance with institutional ethical guidelines and approved by the licensing committee of Kumamoto University (Approval Numbers: A30-051, 2020-099, 2022-077 and A2024-092). Both sexes were considered in the study design and analysis. Three 4-month-old male and female C57BL/6 mice were analyzed in this study. Both liver-specific SerpinA1-overexpressing transgenic (SPA1Tg) mice (8 to 20-week-old male and 12-week-old female) and whole-body SERPINA1 knockout (SPA1KO) mice (11 to 17-week-old male and 12-week-old female) were generated and analyzed by us at Kumamoto University. Adiponectin-CreERT2 mice (10-week-old, male, stock no. 025124) were purchased from Jackson Laboratories. Control (2-month-old male), Ai-IGF1RKO (2-month-old male), Ai-IRKO (2-month-old male) and Ai-DKO (2-month-old male) mice were maintained on a mixed (C57Bl/6 - 129Sv) background by breeding Adiponectin-CreERT2 IRf/f and/or IGF1Rf/f with IRf/f and/or IGF1Rf/f mice, as described in PMID: 28065828 and PMID: 22692545. Control (2 to 3-month-old male), Ai-IRKO (2 to 3-month-old male) and Ai-IRKO without SerpinA1 (2 to 3-month-old male) mice were maintained by crossing Ai-IRKO mice with SerpinA1+/- and IRf/f mice with SerpinA1+/-, both of which were generated by breeding the offspring of Ai-IRKO mice with SPA1KO mice.
Wild animals	This study did not involve wild animals.
Reporting on sex	Male and female mice were used for studies.
Field-collected samples	This study did not involve field-collected samples.
Ethics oversight	ll experiments with research animals were performed in accordance with institutional ethical guidelines and approved by the licensing committee of Kumamoto University (Approval Numbers: A30-051, 2020-099, 2022-077 and A2024-092). Both sexes were considered in the study design and analysis.

Note that full information on the approval of the study protocol must also be provided in the manuscript.

Plants

Seed stocks	*Report on the source of all seed stocks or other plant material used. If applicable, state the seed stock centre and catalogue number. If plant specimens were collected from the field, describe the collection location, date and sampling procedures.*
Novel plant genotypes	*Describe the methods by which all novel plant genotypes were produced. This includes those generated by transgenic approaches, gene editing, chemical/radiation-based mutagenesis and hybridization. For transgenic lines, describe the transformation method, the number of independent lines analyzed and the generation upon which experiments were performed. For gene-edited lines, describe the editor used, the endogenous sequence targeted for editing, the targeting guide RNA sequence (if applicable) and how the editor was applied.*
Authentication	*Describe any authentication procedures for each seed stock used or novel genotype generated. Describe any experiments used to assess the effect of a mutation and, where applicable, how potential secondary effects (e.g. second site T-DNA insertions, mosiacism, off-target gene editing) were examined.*
